# Child welfare worker perspectives on documentation and case recording practices in Canada: A mixed-methods study protocol

**DOI:** 10.1371/journal.pone.0316238

**Published:** 2025-01-07

**Authors:** Nathaniel J. Pollock, Cassandra Yantha, Lil Tonmyr, Kimberly Jewers-Dailley, Melody E. Morton Ninomiya

**Affiliations:** 1 Family Violence Epidemiology Section, Health Promotion and Chronic Disease Prevention Branch, Public Health Agency of Canada, Ottawa, Ontario, Canada; 2 Inuit Tapiriit Kanatami, Ottawa, Ontario, Canada; 3 Community Psychology Program, Wilfrid Laurier University, Waterloo, Ontario, Canada; 4 Department of Health Sciences, Wilfrid Laurier University, Waterloo, Ontario, Canada; University of Perugia: Universita degli Studi di Perugia, ITALY

## Abstract

In health care and child welfare, clinical records and case notes serve multiple functions. When records are aggregated and processed to create administrative data, they can be analyzed and used to inform policy development and decision-making. To be useful, such data should be complete, accurate, and recorded in a standardized way. However, sources of bias and error can impact the quality of administrative data. During the development of national child welfare data in Canada, child welfare sector partners expressed concerns about the accuracy and completeness of data about children and families. This protocol describes a study that seeks to answer two questions: 1) What individual and institutional factors influence how client data is recorded by child welfare workers in Canada? 2) What data quality issues are created through documentation and case recording practices that may impact the use of clinical case management system data for public health statistics? In this protocol, we describe an exploratory mixed methods study that involves an online survey, interviews with a purposive sample of child welfare workers, and a document review of case recording guidelines. To be eligible for the study, participants must have worked at a child welfare agency or department with clinical documentation responsibilities as a part of their job. We will use descriptive statistics to analyze the survey data and thematic analysis to analyze the qualitative data. This study will help uncover strengths, limitations, and possible sources of bias created through case recording and documentation practices in child welfare. Study results will be shared through presentations to interest holders and will inform the further development of national child welfare data in Canada.

## Introduction

In health care and child welfare, clinical records and other forms of case notes written by service providers have multiple functions [1]. These materials document interactions between people and systems of care and record information about patient or client demographics, social and health history, symptoms, diagnoses, and interventions. Such records in written and digital forms also support communication between providers and may form the basis for service remuneration [1]. When information derived from the provision of services or care is aggregated and processed, it becomes administrative data. In the context of public policy, analyzing such data can transform it into evidence for decision-making [2–4].

As a part of a learning systems approach, governments, health and social care organizations, researchers, and communities analyze administrative data to understand population health, describe patterns of service use, and discern how care is being delivered and to what effect [5, 6]. The widespread availability and large scale of data produced by health and social care systems makes it possible to generate evidence in near real-time. Consequently, “big data” have become an increasingly powerful tool in clinical care and policy development [2, 4, 5, 7].

For data to be useful in system-level decision-making, it should be complete, accurate, and recorded in a standardized way [1]. However, administrative data systems are influenced by organizational structures, information management technology, worker practices and perceptions, and legal and policy frameworks [7, 8]. As such, there are several potential sources of bias and error that can be introduced at different points in the “data chain” [1] which can impact the quality and accuracy of recorded information [1, 9, 10]. For example, knowing who can see a case file or how it will be used may affect whether or not information is recorded and the way it is recorded [1, 11, 12].

In child welfare, previous research identified several ways in which biases and errors in case recording may influence administrative data. Biases can operate at the level of individual workers but can also be systemic or structural in nature [5, 7, 11–19]. Child welfare workers may not reliably or comprehensively capture details from interviews in child abuse investigations in case notes [14] especially in when alleged maltreatment is perceived as being less severe [11]. There is also evidence that health and child welfare professionals’ demographic and employment characteristics [20], personal beliefs and opinions [21], implicit biases [10, 22], and racist attitudes [23] can influence assessments [15, 16], decision-making [21, 24], and contribute to disparities in reporting, substantiation, and interventions [18, 25].

At a systems level, a variety of factors can also introduce bias in case recording practices and clinical decisions. Factors include limited training in cultural sensitivity and anti-racist practice [23], a lack of guidelines for case recording [1], caseload size [20], heavy administrative burdens [26, 27], and the tendency to disproportionately investigate racialized [28] and low-income communities [16]. The risk with all forms of bias in case recording is that they become embedded in clinical decision-making [23] and information systems [7, 13] where the cumulative impact may be magnified. In turn, this may lead to inaccurate population statistics that further misinform practice and policy [1, 5, 13].

## Rationale

In Canada, child welfare services are primarily provided by provincial, territorial, and Indigenous government departments or delegated agencies [29]. These organizations are variously referred to as child, youth, and family services, child intervention services, or child and family wellbeing agencies. They deliver a combination of supportive and preventative services, child protection interventions, out-of-home placements, and youth supports [29, 30]. Because child welfare is decentralized in Canada, routinely collected data generated through the delivery of services are primarily held by local or regional organizations [31, 32].

To support national-level monitoring of child welfare indicators, the Public Health Agency of Canada (PHAC) collaborated with partners to develop an administrative database called the Canadian Child Welfare Information System (CCWIS) [31]. As a source for statistical analysis, the CCWIS includes population-based data about families who have contact with the child welfare system, such as children placed in out-of-home care [31]. In recognition of the possible limitations, biases, and other data quality issues (e.g. missing data) that are common with administrative data [1, 4, 33, 34], CCWIS data may be strengthened by understanding the processes and contextual conditions which generate these data. This understanding may help during analysis and interpretation [1], and can inform case recording practices and information system design and management.

A principal assumption and part of the genesis of this project is that administrative data has systemic errors and biases [34, 35]. A challenge is that errors and bias may go undocumented or unacknowledged. For example, missing data is a common problem in epidemiologic research [36] and in child welfare research [37]; the reasons data are missing may be both unknown and not random [36, 37].

Throughout discussions about developing national child welfare data in Canada, PHAC consistently heard concerns from child welfare sector partners about challenges related to data accuracy and completeness [38]. For example, similar to prior research [39], partners reported that Indigenous identity (that is, whether a child in contact with child welfare services identifies as First Nation, Inuit, or Métis) is not consistently recorded in client records, and therefore when data about Indigenous children and youth are reported, it may not be accurate [40]. At a systems level, inaccuracies in data about identity and demographics are problematic because they limit opportunities for equity-oriented population health assessment. In Canada, this limitation in particular makes it difficult to address gaps in data about First Nations, Inuit, and Métis children and youth, such as those highlighted by the Truth and Reconciliation Commission (TRC).

In 2015, the TRC underscored the need to report on key indicators about Indigenous children and youth, especially those in out-of-home care [41]. This need was included among 94 Calls to Action delivered by the Commission to redress the historical and ongoing impacts of colonization on Indigenous Peoples in Canada, including the overrepresentation of Indigenous children in the child welfare system. In this context, understanding the factors that influence the quality of child welfare data early-on in the data cycle may contribute to efforts to improve case recording practices and in turn, better equip decision-makers with trustworthy evidence.

### Research questions

This study is guided by two research questions: 1) What individual (e.g. implicit biases, training, beliefs and attitudes, experiences) and institutional (e.g. caseload size, recording forms, data linkages) factors influence how client data is recorded by child welfare workers in Canada? 2) What data quality issues are created through documentation and case recording practices that may impact the use of clinical case management system data for population-level statistics?

### Objectives

This study aims to reveal how and what information child welfare workers document about children and families, and if workers have suggestions about how to improve the quality of case recording. The objectives of the study are to:

1. describe the documentation and case recording practices of child welfare workers;
2. understand the contexts and circumstances of documentation and recording that may impact the quality of administrative data in child welfare; and
3. identify strategies for increasing data quality and reducing potential bias in child welfare administrative data.

### Theoretical frameworks

Our research questions, approach, and tools draw on theoretical frameworks from institutional ethnography (IE) [42, 43] and integrated knowledge translation (IKT) [44–46] to explicate, examine, and reveal the decisions and practices that underpin case recording and documentation in child welfare. While this study is not using IE as method of inquiry, we will explore how institutional structures, policies, and texts shape the everyday activities and documentation practices of child welfare workers. By mapping the connections between workers’ experiences and the broader organizational context, we can begin to uncover the institutional influences that may contribute to data quality issues in epidemiological analyses with administrative data.

In parallel, the framework of IKT guides our approach to engaging interest holders and partners, particularly child welfare workers and public health data users, as active collaborators in the research process. By incorporating their insights, the findings and recommendations for improving documentation practices will be grounded in real-world contexts that may be directly applicable to enhancing data quality and awareness of the extent of existing biases in child welfare data. This application of an IKT framework not only deepens our understanding of institutional factors at play but also ensures that the research outcomes are both meaningful and actionable.

## Methods

This exploratory, sequential mixed methods study [47] involves three phases: 1) an online survey; 2) interviews with a purposive sample of child welfare workers; and 3) a document review to describe case recording guidelines and understand the contexts in which clinical information is documented in child welfare. The survey will support a descriptive analysis of existing practices in the field [48]. The subsequent qualitative interviews will allow for an in-depth exploration of the contextual and experiential factors influencing these practices [49]. The document review will add another layer of understanding by examining the formal guidelines and requirements that shape documentation behaviors. Integrating these three methods will help show not only what is happening in practice but also why it occurs, how policies and procedures coordinate how people record data, and how practices align with or diverge from formal policies [50–52].

This study was approved by PHAC’s Science Review Committee, underwent a Health Canada/PHAC privacy impact assessment, and was approved by the Health Canada/PHAC and Wilfrid Laurier University Research Ethics Boards (REB 2024-002P and REB 8935, respectively).

### Partnerships and researcher characteristics

Collaborative relationships between researchers, decision-makers, and practitioners can help facilitate project development and design, the selection of data collection sites, research licensing, participant recruitment, feedback on data analysis and interpretation, and dissemination and uptake of study results [21, 45, 53, 54]. At present, this study includes a partnership between a federal government agency, a university-based researcher, and Inuit Tapiriit Kanatami, the national Inuit organization in Canada. Through the recruitment process, this project may develop additional partnerships with child welfare and child wellbeing agencies, provincial/territorial government departments, and other organizations.

The research team includes three investigators [NJP, CY, and LT] who have postsecondary education in social work and have worked in the child welfare system; four team members [NP, LT, KJD, MMN] have graduate-level training in sociology, epidemiology, psychology, community health; one investigator [CY] has a child welfare policy position in a national Inuit organization, two [NP, LT] conduct public health surveillance and research at the national public health agency, and one [MMN] holds a university research chair position in community health and knowledge mobilization. Four team members [NP, CY, LT, and MMN] have professional networks that include child welfare organizations from across Canada. All research assistants conducting interviews in this study will have extensive experience in qualitative research.

### Study population and sample size

The precise sampling frame for survey participants cannot be determined a priori as the agencies and departments responsible for child welfare services in each province and territory vary in size. Relatedly, national data on the child welfare sector workforce is limited because of differences in professional requirements related to education and training. Child welfare services are provided primarily by social workers, though in some jurisdictions, educational qualifications vary. In Ontario for example, a study found that 30% of workers with investigative roles had educational backgrounds in fields other than social work [55]. Nonetheless, social work is the primary profession in child welfare practice [26, 55].

In 2021, there were 58,159 social workers in Canada [56]. A 2018 survey of social workers (n = 3,258) conducted by the Canadian Association of Social Workers reported that 53% of respondents worked in child welfare [26]. Although the survey used a convenience sample and so may not be generalizable, if the results were representative, there could be upwards of 24,000 social workers in child welfare in Canada. Further, the potential pool of eligible participants for our study would also likely exceed this number because those who previously worked in child welfare and those who are not social workers but work in a child welfare role are also eligible.

Our goal is to recruit 25 to 35 child welfare workers to participate in an interview. This recruitment target is sufficient for achieving both precision and “information power” [57] in the results because the objectives of our study are narrow and descriptive, and our sample population is specific [57]. Previous qualitative [23, 27] and mixed-methods studies [54] with child welfare professionals recruited a similar number of participants for interviews (range: 27 to 34). Based on this target for interviews and the assumption that 10% of a convenience sample will agree to an interview, we propose to recruit between 250 and 350 survey participants. Given that the study objectives are exploratory with no intent to produce generalizable statistics, the sampling frame is large, the participants are accessible and have specialized knowledge, and that we are using mixed methods to triangulate the results, these recruitment targets are practical and sufficient for producing meaningful data.

### Eligibility criteria for participants

To be eligible for the study, participants must be currently or recently employed within the past two years, by a child welfare agency, department, or organization; have a “front-line” role or position that involves regular client contact, service or case management, or program delivery activities; and have documentation responsibilities as a part of their job. This includes social workers and child welfare workers who do not have post-secondary education in social work. We will exclude participants if they do not meet eligibility criteria for participation due to their employment role. For example, those who work as administrative staff, supervisors, managers, directors, or are primarily responsible for data management and analysis.

For the interview phase of the study, we will recruit a purposive subsample of 25–35 participants. Potential interviewees will be selected from the pool of survey respondents. To obtain a heterogenous sample, we will seek participants with varied training/educational backgrounds (e.g. social work, social services worker, social sciences, psychology, child and youth care, and community studies), credentials (registered social worker, other), roles (child protection, in care, youth services, family support), number of years in practice or career stage (early, mid, and late career), gender, race/ethnicity (Indigenous, other racialized group, White), age, and from various regions of Canada (Atlantic, Eastern, Prairie, Western, and Northern).

### Recruitment

Our recruitment process will begin in September 2024 and use two parallel strategies: 1) partnering with a purposive sample of child welfare agencies and 2) inviting child welfare staff through professional bodies and relevant networks. In our first strategy, we will seek to recruit data collection sites from diverse rural, suburban, and urban locations across Canada, including regions that serve predominantly Indigenous and/or racialized communities or populations. Recruiting departments/agencies will involve using pre-existing relationships and networks to advertise the study to decision-makers in child welfare government departments and agencies. Our team’s ongoing involvement in child welfare research, data, and policy means that we have established partnerships with many possible data collection sites. Given the nature of child welfare practice, it may be difficult to recruit child welfare system partners without such relationships. However, these relationships may also not be sufficient as child welfare agencies and departments may have limited research capacity [58] or low interest relative to service delivery and other priorities [59].

For partnering agencies/departments, we will send an email with a poster invitation, study information with consent form, survey, and interview guide. Each site will decide how to best circulate the email invitation. It is anticipated that the participating sites will circulate an email with a link to the survey and a recruitment poster that briefly describes the study to all eligible participants.

Our second recruitment strategy will involve professional and relevant networks. We will invite eligible participants through national and provincial/territorial professional bodies, agency associations, post-secondary regulators, and/or accreditation bodies related to child welfare or social work. The dissemination of the study advertisement will be done through online sources such as social media, membership listservs, and via email. Distributing a notice about the study in this way allows us to reach eligible participants irrespective of their employer’s relationship to the study. We will monitor which agencies, regions, and geographies are represented by survey respondents.

After one month of circulating invitations, we will send a reminder email to all of the people and groups we reached out to initially. Two months after initiating recruitment, if there are underrepresented regions (e.g. provinces and territories) or types of child welfare agencies (e.g. urban/rural/remote, close to Indigenous communities), we will let contacts within social work organizations, child welfare organizations and networks, regulation authorities, departments and agencies know we are looking for stronger representation and ask how to best recruit more participants.

Study information and informed consent will be available online within the survey platform before participants begin completing the survey. Similar to a previous Australian study with child welfare professionals [21], we will recruit interview participants from the pool of survey respondents. At the end of the survey, respondents will be asked if they are willing to be contacted about participating in an interview. Based on the demographics of survey respondents that indicate willingness to be contacted about an interview, the research team will invite potential interview participants based on demographic diversity (e.g. gender, age, years of experience, race/ethnicity). This purposive sampling strategy supports the inclusion of a heterogenous population.

### Consent process

For survey participants, informed consent will be obtained through the online survey platform before proceeding to the survey questions. At the end of the survey, participants will be asked if they are willing to participate in an online or in-person (if feasible) interview. If they are willing, participants will be asked to provide their name and preferred email address. Prospective interview participants will receive a copy of the consent form and interview guide via email so they have an opportunity to review the material in advance.

Before the interview begins, the interviewer will verbally review the consent form with the participant to provide an opportunity to ask questions and clarify any details. Participants will be given the option of providing audio-recorded verbal consent or consent using the video conference chat feature. Data generated by the study described in this protocol will only be available to the research team; this measure is in place to protect the privacy and confidentiality of participants. Access to the data for secondary analysis will only be considered on a case-by-case basis due the risks to privacy and confidentiality of participants and federal privacy laws.

### Data collection

Data collection for this study will involve three components: 1) an online survey; 2) interviews; and 3) a document review. Quantitative and qualitative information about participant demographics, employment and agency/department characteristics, and child welfare data will be collected through a survey in Qualtrics (Provo, Utah, USA). Qualtrics is a secure online survey platform that is programmed to be user-friendly on a desktop computer or a mobile phone. While Qualtrics has the built-in capacity to detect and identify potentially fraudulent responses for researchers, we purposefully included mandatory questions within the survey for validation in case we suspect fraudulent survey responses (i.e. bots or people hoping to receive a draw prize).

Survey questions are designed to gather respondent demographic information such as years of experience, age, gender, training, workplace region, and cultural/racial identity; aspects of recording practices such as the conditions in which recording happens, information that may be helpful but is not requested, information that is awkward or often skipped; and views on the use of statistics from child welfare data. The survey is intended to take participants approximately 15 minutes to complete and can be completed in English or French. All survey respondents may enter their name for a chance to win one of five prize draws of $50 e-gift certificate. The survey questions are available in S1 File.

In-depth qualitative data will be collected through interviews. Interviews will be conducted in a setting that is agreed upon by the participants and interviewer, which may include video or audio call or be in-person, in English or French; the estimated time to complete the interview is 30 to 45 minutes. Interviews will be audio-recorded and the recordings will be transcribed verbatim. We will ask validating questions during the interview and if a participant’s interview response does not match their survey response, we will not include their survey or interview data for analysis. All interview participants will receive a $25 e-gift card as a recognition of their participation. The interview guide is available in S2 File.

Data will also be collected through document review. The principal investigator may seek or request access to training materials, documentation, and policy and procedure manuals mentioned in surveys and interviews. S3 File includes a list of the types of documents that will be requested. All data collection activities will be conducted by the PI and/or an experienced research assistant under the PI’s supervision. Individuals who participate anonymously will not be given the option to withdraw their data after completing the survey. However, interview participants may withdraw or terminate their participation at any time and request that their data be removed from the quantitative and qualitative analysis for up to one month after their interview.

We will link demographic information (i.e. gender, location, years of experience, cultural/racial identity) to each interview transcript for analytical purposes. A digital key that links interview participant names, email, transcript, survey response, and relevant documents that might be shared, will be saved in a password-protected file that only one co-PI [MMN] will be able to access. Raw data, processed analytical files the study key, and preliminary findings documents will be saved on a secure server at Wilfrid Laurier University and will require a minimum of two layers of password-protection.

### Data processing and analysis

For the quantitative analysis, we will export all data from Qualtrics and import it into SPSS. Data will be removed if the responses are 1) suspected to be fraudulent or 2) if people do not answer the demographic questions and answer less than 50% of the remaining survey. After all names and email addresses that are linked to unique response IDs are removed, the data will be cleaned. Descriptive statistics including frequencies and proportions will be used to summarize the quantitative data. We will summarize the data and conduct stratified analyses to examine how individual or intersecting identities (e.g. gender, cultural/racial identities, age) of child welfare staff relate into documentation practices and attitudes. After descriptive analyses have been completed, we will assess the feasibility of conducting additional analyses to examine relationships between demographics, work-related factors, and documentation practices.

When we report on specific patterns or trends, we will also report on and discuss contextual considerations such as regions where there are disproportionate numbers of Indigenous and/or racialized children and families involved in child welfare and staff are predominantly White. To protect the privacy of individuals and agencies, we will ensure that data are sufficiently aggregated, and cell counts of less than five will be suppressed. Survey findings will not be presented in a way that offers enough information to identify any participants. Narrative data from open-ended questions in the survey will be analyzed using thematic analysis as described by Braun and Clarke (2006) [60].

Interview transcripts will be analyzed in NVivo, a qualitative/mixed methods analytical software. First, two team members will independently familiarize themselves with the data by reading through all of the transcripts. Second, the same two people will randomly select 10 to 12 transcripts and independently code the transcripts for relevant information. Third, they will independently collate codes into potential themes and then compare and discuss themes with the aim of reaching consensus on codes and potential themes. Fourth, two researchers will then re-code the 10–12 previously coded transcripts in NVivo, checking for inter-coder reliability using Cohen’s Kappa of 0.7 or higher. If the level of agreement is below 0.7, the two researchers will discuss and resolve the differences in their coding, refine the codebook, and independently code another six transcripts that were not previously coded. This process will continue until we reach a score of 0.7 or higher. All transcripts that were not part of the transcripts included with a high level of agreements (i.e. Kappa > 0.7) will be (re-)coded by one of the two researchers. Any new codes or emergent themes that arise after analyzing all of the transcripts will be discussed with the aim of reaching consensus between the two researchers analyzing the transcripts. Fifth, both researchers will collaboratively review all coded texts for each theme will be analyzed further to define with clear examples [61], perhaps re-name, and tell a story. Sixth, the preliminary findings will be shared back with participants as a form of member checking [62], using a webinar format in Zoom to present the findings and attending participants can make anonymous or private comments or ask questions. All interview participants will also be invited to contact researchers for a one-on-one call to review or discuss the preliminary thematic findings. We will also present preliminary findings to key interest holders and participating agencies/departments for feedback, comments, and questions that may lead to further analysis.

The findings will be organized around three broad areas: 1) the conditions and realities in which staff record data; 2) personal data recording practices that reveal challenges, discomforts, limitations, and training background; and 3) child welfare administrative data perceptions and observations about its value, use, reliability, potential biases, and limitations.

Documents mentioned in the online survey and interviews will be gathered and reviewed. If documents are not available from the public domain, we may not be able to include them in the analysis. We will review documents in NVivo using codes and memos. We will triangulate the codes and memos from the documents with findings from the survey, interviews, and literature review to identify how the documents dictate, inform, or organize how child welfare workers record data.

### Techniques to ensure trustworthiness and credibility

In accordance with methodological guidelines for qualitative and mixed methods research, we will use multiple methods to maximize trustworthiness and credibility [47, 60]. At the study design phase, the data collection tools and analytical methods were reviewed and piloted by multiple researchers with expertise in the methods and field, as former child welfare workers. All survey analysis will be conducted by two researchers independently and findings will be compared to maximize internal validity.

The interviews will be conducted by research team members who have no conflicts of interest with participants; interviewees will have an opportunity to add, edit, or clarify information shared during their interview through a member checking process (i.e. review their transcript before analysis); we will make our research methods transparent in publications and reports; all interview data will be thematically coded independently by two researchers before being discussed and consensus is reached on an analytical code book for the complete analysis. Only the last author (MMN) and research team members without any conflict of interest (if any arise) will have access to raw data that may identify participants.

We will triangulate the quantitative survey findings, qualitative interview findings, and document analysis to provide a comprehensive understanding of the child welfare data case recording practices. By comparing and contrasting findings from these three data sources, we can identify consistencies and discrepancies, strengthening the validity and credibility of the results [63]. This process will involve synthesizing quantitative results to reveal patterns, interpreting qualitative data to explore underlying reasons and meanings, and analyzing documents to contextualize and corroborate findings within established practices or policies [64]. The convergence of evidence from these diverse methods offers a more nuanced and robust understanding of child welfare recording practices [65].

### Strengths and limitations

Strengths of this study include the use and integration of multiple data sources (survey, interview, and document review) to understand case recording practices. Comparing responses between survey and interviews also allows us to assess agreement, which increases the credibility of the findings. Pre-existing relationships between the Public Health Agency of Canada, Inuit Tapiriit Kanatami, and child welfare agencies/departments make this project both feasible and possible. These networks will be useful in helping to recruit participants to the study and for dissemination.

A limitation of this study is that we will use a purposive sample for the survey that may not be representative of all child welfare workers in Canada. There is an inherent risk of bias within the sample population since child welfare workers are not required to complete the survey. In other words, survey respondents and specifically survey respondents that indicate willingness to participate in an interview may be inherently motivated to participate out of concern and interest in how data are, or are not, recorded. While we hope to reach perceived data saturation with 25 to 35 interview participants, it is possible that this will not happen. Due to the sampling methods and design, the results of this study will not be generalizable or representative of the population.

Another limitation is that leadership and information management staff within child welfare agencies are not eligible to participate if they do not otherwise have roles in front line service delivery, even though they may have valuable perspectives on data quality and case recording practices. Including leadership and management staff who are not directly involved in data recording would require asking different questions and may make participants identifiable. An additional limitation is that participants may feel uncomfortable with survey or interview questions that ask about practices and training related to gender, race, Indigeneity, and discrimination [22]. This may in turn influence responses. Further, the recruitment of interview participants from the pool of survey respondents may create a selection bias, as child welfare workers who are most motivated, such as those who have more negative experiences or views about administration burden, may be more likely agree to be involved.

### Expected outcomes

Access to administrative data can allow public health practitioners, researchers, and policy makers to develop, inform, and monitor efforts to positively impact the lives of children and families who have contact with child welfare systems [32]. By understanding the processes and conditions which produce data about children and families, we may uncover strengths, limitations, and possible sources of bias created through the process of case recording and documentation in child welfare. This evidence can support the transparent use of administrative data for performance measurement and population-level statistics, and may serve to guide quality assurance initiatives and training to promote evidence-informed practice [66]. The results of this study will also be applied to PHAC’s work related to the ongoing development and improvement of national child welfare data in Canada [31].

### Integrated knowledge translation and dissemination plan

This study uses an integrated knowledge translation approach to the design and dissemination of results [67]. Data collection tools and preliminary findings will be shared with child welfare partners and interest holders for feedback and input. We will invite participants to suggest ways in which they would like to receive the final results from this study through the survey and interviews.

In addition, summary findings will be disseminated to public health and child welfare audiences through the existing networks of study investigators. For example, PHAC’s epidemiological work related to child welfare is guided by the national Child Maltreatment Surveillance and Research Working Group. This working group is comprised of leading Canadian scientific experts, national Indigenous organizations, representatives from provincial/territorial child welfare departments, practice and policy consultants from child welfare, and representatives from various federal departments (statistics, justice, Indigenous services, and public health) and other national health data-related organizations [31]. We will share the final results with this working group, and with contacts and networks, through presentations, a summary report, and a scientific manuscript. Involving knowledge users and interest holders in the development of this study and the interpretation of the results, and using varied knowledge sharing strategies that are tailored for key audiences, may increase the uptake and application of the study findings [68].

## Conclusion

The information about children and families that is documented by child welfare workers in case notes form the basis of the administrative data that are used in population statistics, research, and performance measurement in child welfare and public health. Such data are a valuable resource and can be analyzed to generate evidence for practice and policy decisions. To understand the quality of administrative data in child welfare and gain insight into the potential sources of bias and error that may become embedded in data during the case recording process, it is necessary talk to front line staff who are responsible for generating case records.

## Supporting information

S1 File Survey questions. (DOCX)

S2 File Interview guide. (DOCX)

S3 File Types of documents requested. (DOCX)
