# Supplementary material for: Child welfare worker perspectives on documentation and case recording practices in Canada: A mixed-methods study protocol
Source: PLoS One. 2025 Jan 7;20(1):e0316238. doi: 10.1371/journal.pone.0316238 (PMC11706400; doi:10.1371/journal.pone.0316238)
Supplement: S1 File — (DOCX) [file pone.0316238.s001.docx]

**SURVEY QUESTIONS**

1. **Work Setting Demographics (REQUIRED QUESTIONS)**
   1. Do you currently work in the field of child welfare, child protection, child and family services, or for a child and family wellbeing or services agency?
      - If no, have you worked in this field in the past two years?
   2. What child welfare agency (or department) do/did you work for?
      - Free text _____
      - Prefer not to respond
   3. What is the geographic setting for the catchment area where you work or worked most recently? (Select all that apply)
      - Urban
      - Rural
      - Northern
      - Remote
      - Other (Please specify: ___________)
      - Unsure
      - Prefer not to respond
   4. What is/was your role at this agency or department?
      - Child protection
      - In care
      - Youth services
      - Family support
      - Other (Please specify: ___________)
      - Unsure
      - Prefer not to respond
   5. What is your employment status in your current or most recent position?
      - Permanent
      - Contract
      - Other (Please specify: ___________)
      - Unsure
      - Prefer not to respond
   6. Approximately, how many years have you worked...
      - at this agency/department? ____
      - in this position? ____
      - in the field of child welfare? ____
      - Prefer not to respond
   7. What is your educational background? Select all that apply.
      - Social Service Worker Diploma
      - Child & Youth Care Diploma
      - Bachelor of Social Work
      - Bachelor’s degree in a field other than social work
      - Master of Social Work
      - Other (please specify): _____________
      - Prefer not to respond
2. **Workload and Documentation**

For participants who no longer work in child welfare, answer the questions based on your most recent experience in the field.

- 1. How many families do you have on your caseload right now?
     - Narrative for details:
     - Does not apply
     - Prefer not to respond
  2. Briefly describe the population of clients you work with.
     - Narrative for details:
  3. In an average week, what % of time do spend on the following? (MATRIX QUESTION)
     - Direct client contact ( ___%)
     - Documentation/case notes ( ___%)
     - Collateral contacts or other professionals or persons in the circle of care ( ___%)
     - Other. Please list activity: ____________ ( ___%)
     - Other. Please list activity: ____________ ( ___%)
  4. What is the name of the client information/case management system used at your office? _____
  5. What do you see as the main purpose(s) of recording client information or case notes (in a few words)?_____
  6. To what extent do you record information about client strengths (Likert scale: “not at all à very”)?
  7. Do you ever want to record information that does not fit into the defined fields or drop down menus? If “no”, skip.
     1. If “yes”, have follow up question: What do you do? (Drop down menu of “select all that apply”: I do not record it (assume it will not be looked at or be relevant); I work it into my notes; other: please specify _________
     2. How often do you have information you want to record but it does not fit into defined fields or drop-down menus? (Likert scale “not at all à very”, followed by a text box for examples).
     3. What type of information (e.g. field) do you wish had more room for details or was recorded at all in your data system? [text box]
  8. Do you routinely skip or avoid recording information? If “no”, skip.
     1. If “yes”, why? [Check all that apply: too awkward or inappropriate to ask, takes too long to record, not important, do not understand what is required, protecting children and/or family members, other: ___________]
  9. How do you typically determine the following demographic information about clients? (Check 1 per factor)

| **Demographic factor** | **Ask clients directly** | **Look at official documents** | **Based on observation** | **Guess** | **Do not record this** | **Other (specify)** | **Prefer not to respond** |
| --- | --- | --- | --- | --- | --- | --- | --- |
| Age |  |  |  |  |  |  |  |
| Sex (assigned at birth) |  |  |  |  |  |  |  |
| Gender, including Trans and non-binary identities |  |  |  |  |  |  |  |
| First Nations, Inuit, or Metis identity |  |  |  |  |  |  |  |
| Racial, ethnic, or cultural identity |  |  |  |  |  |  |  |

- 1. Based on your experience, what is a developmentally appropriate age to be asking children/youth questions about identity (e.g. gender, Indigenous identity, etc.)?
  2. Have you received training while at your current agency/department related to: (MATRIX QUESTION)

| Training Topics | Required | Encouraged | Sought it out myself | Never trained | Do not recall | Prefer not to respond |
| --- | --- | --- | --- | --- | --- | --- |
| 2.7.1 Documentation and recording client information practices |  |  |  |  |  |  |
| 2.7.2 Gender-affirming care |  |  |  |  |  |  |
| 2.7.3 Trauma-informed care |  |  |  |  |  |  |
| 2.7.4 Indigenous cultural safety |  |  |  |  |  |  |
| 2.7.5 Anti-oppressive or anti-racist practice |  |  |  |  |  |  |
| 2.7.6 Disability-informed practice |  |  |  |  |  |  |
| - - 1. Child welfare statistics, performance measurement, or data analytics |  |  |  |  |  |  |

- 1. Approximately how much total training have you received on how to use the client information/case management system?
     - None
     - Less than 2 hrs
     - 2-4 hrs (1/2 day)
     - 5-10 hrs (full day)
     - 11-20 hrs (2-3 days)
     - 21+ hrs (more than 3 days)
     - Prefer not to respond
     - Comment:

1. **Perspectives on Child Welfare Statistics**
   1. When you see statistics about child welfare services, such as the number or rate of children in care, what is your usual reaction to them?
   2. Where would you look for trustworthy statistics about the child welfare system, such as the number or rate of children in care? Check all that apply.
      - 1. my agency
        2. scientific literature
        3. government websites
        4. stakeholder/advocacy groups
        5. Other (please specify): ______
        6. Prefer not to respond/disclose
   3. Rate your responses to the following questions from “not at all à very”, plus “prefer not to disclose”. (MATRIX QUESTION)
      - 1. In your view, how are accurate are child welfare statistics? (5-point Likert scale)
        2. How trustworthy are child welfare statistics? (5-point Likert scale)
        3. How interested are you in understanding more about child welfare data and statistics? (5-point Likert scale)
   4. What are some reasons that child welfare statistics might be inaccurate?
      - Narrative response:
      - Prefer not to respond
   5. What ideas do you have for how to improve child welfare data and statistics?
      - Narrative response:
      - I do not think child welfare data and statistic need to be improved.
   6. What kinds of statistics about child welfare would be most useful to in your clinical practice or for your agency?
   7. What kinds of statistics about child welfare would be most useful to provincial/territorial, Indigenous, or federal policy?
   8. Did you take a statistics course during your post-secondary program (e.g. undergraduate degree, diploma, etc.)? (Yes, no, don’t remember, prefer not to answer).
   9. How comfortable are you with understanding and interpreting statistical information from: (5 point Likert scale “not at all à very”)
      - 1. Your agency/department such as monthly or annual reports or statistics
        2. Government reports
        3. Journal/research articles
   10. Is there anything else you would like to let us know?
2. **Personal Demographics**
   1. Where do you live (province/territory from list)? _____
   2. Which best describes your gender? Check all that apply.
      - Woman
      - Man
      - Nonbinary
      - Questioning/Exploring
      - Gender identity not listed (please specify): ___________________
      - Prefer not to respond
   3. Which cultural and/or racial identity do you identify with? Check all that apply.

- White
- South Asian (e.g., East Indian, Pakistani, Sri Lankan)
- Chinese
- Black
- Filipino
- Indigenous (e.g. First Nation, Inuk/Inuit, or Métis)
- Arab
- Latin American
- Southeast Asian (e.g. Vietnamese, Cambodian, Laotian, Thai)
- West Asian (e.g., Iranian, Afghan)
- Korean
- Japanese
- Other — specify: [open text]
- Unsure
- Prefer not to respond
  1. How old are you? (Select one): 20-24, 25-29, 30-34, 35-39, 40-44, 45-49, 50-54, 55-59, 60-64, 65-69, 70+ years, Prefer not to respond
  2. In recognition of your participation in this survey, you can choose to be entered into a draw for a $50 gift card. Do you want a chance at winning a $50 gift card?
     - Yes/No
  3. For the next part of this study, the research team will be conducting 1:1 interviews with child welfare workers to learn more about how client and case details are documented and recorded in case notes and information systems. For example, we will ask questions about factors that influence documentation, what kinds of information is included in case notes, and about the role of statistics in child welfare. Would you like to participate? (Yes, no). If you answer “yes”, you may be invited to participate in a 1:1 virtual interview with a member of the research team. All interview participants will receive a $25 e-gift card as an expression of appreciation. Your survey responses will be linked to your interview responses. This will help us understand your work and experiences, and reduce the number of questions we need to ask.
     - Yes/No
  4. If you answered “yes” to one or both of the above questions, we need your name and preferred email address. Your name and contact information will be kept private. We will only use your name and email address for the prize draw and/or invite you to participate in an interview. If you participate in an interview, your survey responses will be connected to your interview responses. This will help reduce repetition and the time it takes to be involved in the study. You can participate in the survey and decline the interview without any consequence; both are optional and voluntary.
     - Last Name:
     - First Name:
     - Preferred Email address:
