# Supplementary material for: Child welfare worker perspectives on documentation and case recording practices in Canada: A mixed-methods study protocol
Source: PLoS One. 2025 Jan 7;20(1):e0316238. doi: 10.1371/journal.pone.0316238 (PMC11706400; doi:10.1371/journal.pone.0316238)
Supplement: S2 File — (DOCX) [file pone.0316238.s002.docx]

**INTERVIEW GUIDE**

1. **Introduction**
   1. Please tell me a bit about your work. What are the areas or types services that you are responsible for?
2. **Experiences with documentation in child welfare**
   1. What is your process for documenting clinical/client information?

- Timing – when during the day/week do you typically do this?
- Location – where do you do it?
- Paper vs computer?
- Is editing your notes possible? If so, how often do you do this?
  1. What are the most important kinds of information to document?
  2. How does your documentation process compare to your colleagues?
     - *Prompt:* Is your approach similar?
  3. Who sees the information that you record?
  4. How is recorded information used? And how does [*answer to how info is used*] inform what you document?
  5. What laws or policy/procedures have the biggest impact on your documentation practices?
  6. What are some challenges that you or your colleagues experience with documentation? Probe: Does feeling stretched or burnt out affect your feelings and practices related to documentation?
  7. Did COVID-19 impact your overall experiences with documentation? If “yes”, how so?
  8. What training do you receive on how to document/record client information?
  9. What accuracy checks are there to make sure that data is being recorded properly? Are there different checks for demographics and clinical information?
  10. Do you have training manual or a guidebook (with documentation standards) that you consult when you are not sure what you are required to record, and how much information to include?

1. **Recording practices related to client information in child welfare**
   1. When you meet a client/family for the first time, how do you record information about their circumstances? Please describe how you usually approach this part of your work.
   2. What fields do you wish had more room for details or was recorded at all in your data system?
   3. Have you ever noticed errors in a client’s record in the information system? If so, what kinds, and how do you deal with them, if at all?
   4. Are any details routinely missing from client records? If so, which types of information?
   5. Is there anything you routinely skip or avoid in your documentation?
   6. Have you ever recorded information or been asked to record information that you felt uncomfortable recording?
      - If so, what can you say about this?
   7. How do you determine sex and/or gender identity of clients?
      - 1. How do you record it in the data system?
   8. How do you determine cultural/racial/Indigenous identity if clients?
      - 1. How do you recorded it in the data system?
   9. How does documentation get handled if/when you aren’t sure about a client’s identity or the client does not want to disclose it too you?
   10. How do you account for things like age, gender, or ethnicity in your work with clients? In other words, do you do things differently, depending on people’s age, gender, or ethnicity? If so, what?
2. **Understanding and improving child welfare statistics**
   1. How does your agency/department use data or statistics?
      - What does it use them for?
   2. What statistics does your employer share with staff? [Prompt: AGM report, monthly stats, research, etc.]

Preamble: Documentation and record keeping can serve many purposes including generating statistics that may inform decisions and resource allocation.

- 1. What concerns, if any, might you have about the accuracy of statistics?
  2. Any ideas on how to improve the quality and accuracy of data? [Probes: more time, different fields/recording methods, training on use of statistics.]
  3. If you knew client data that you recorded was going to being used for research by external partners such as a provincial or federal government department or a university researcher, is there anything you would change about what and how data information is recorded and shared? Explain.
  4. How would you like to receive the findings from this study? [Share a list of planned deliverables and invite other ideas, if they have any.]
