# Supplementary material for: Child welfare worker perspectives on documentation and case recording practices in Canada: A mixed-methods study protocol
Source: PLoS One. 2025 Jan 7;20(1):e0316238. doi: 10.1371/journal.pone.0316238 (PMC11706400; doi:10.1371/journal.pone.0316238)
Supplement: S3 File — (DOCX) [file pone.0316238.s003.docx]

**DOCUMENT REVIEW**

The principal investigator will review materials posted on government department or agency websites related to documentation and recording practices. For any participating agencies, these documents will be requested for the document review component. This list may be updated to include documents or training identified during focus groups/interviews.

In the meetings with the agency’s main contact person, they will be asked:

1. What is the database system that is used to track client information?
2. Is there a codebook available (description of variables to collect)? Training guide for employees involved in data collection? What variables are mandatory or optional?
3. Can you provide copies of recent reports that were generated using data from the child welfare database?
4. Can you share any documents/forms (digital or paper) that staff frequently use that are entered into the database later? Probe about documents mentioned in focus groups and/or interviews.
